# Soil and Water Pollution in Latin America and the Caribbean: A Systematic Review of Impacts on Ecosystems and Public Health

**DOI:** 10.1155/jt/5181162

**Published:** 2025-10-30

**Authors:** Danladi C. Husaini, Jennesa Oh, Megli Perez, Joel H. Chiroma

**Affiliations:** ^1^Allied Health Department, Faculty of Health Sciences, Pharmacy Unit, University of Belize, Belmopan, Belize; ^2^Faculty of Nursing, University of Abuja Teaching Hospital, Gwagwalada, Abuja, Nigeria

**Keywords:** bioaccumulation, ecosystem health, heavy metals, Latin America and the Caribbean, pesticides, public health, qualitative synthesis, soil pollution, systematic review, water pollution

## Abstract

**Background:**

Latin America and the Caribbean (LAC), a region of critical biodiversity and natural resources, faces escalating threats from anthropogenic soil and water pollution. While individual studies have documented contamination, a comprehensive synthesis of its impacts on ecosystems and public health across the region was lacking.

**Objective:**

This systematic review aimed to map and qualitatively synthesize the empirical evidence on the impacts of soil and water pollution in LAC, identifying regional research trends and critical knowledge gaps.

**Methods:**

A systematic search was conducted across five databases (EBSCOhost, PubMed, SciELO, JSTOR, and HINARI) for peer-reviewed literature (2008–2023). Following a blinded, two-phase screening of 1145 records via Rayyan software, 11 studies met the predefined inclusion criteria for qualitative synthesis. Data on geographic context, pollutant profiles, exposure assessment, and health/ecological endpoints were extracted and analyzed descriptively.

**Results:**

The evidence base exhibited a pronounced geographical skew, with 8 of 11 studies originating from Brazil, and no eligible studies from Central America or the insular Caribbean. Synthesis of human studies revealed pervasive subclinical health effects, including respiratory symptoms in ≤ 40% of agricultural workers, endocrine disruption strongly correlated with organochlorine exposure (*r* = 0.68–0.72; *p* < 0.001), and cholinesterase inhibition in 63.8% of organophosphate-exposed subjects. Aquatic systems showed widespread contamination, with pesticide and metal concentrations spanning 0.0047 μg/L to 2110 μg/L. Herbicides dominated contaminant profiles, with compounds like clomazone detected in 100% of river samples, while metals including lead demonstrated clear bioaccumulation in fish muscle. Several studies identified latent ecological risks, with arsenic carcinogenicity risk > 10^−6^ and herbicide mixtures endangering aquatic biota.

**Conclusions:**

Despite the absence of acute poisoning events, the convergence of evidence signals a latent threat from the pervasive, low-level contamination of LAC's environments. The documented subclinical impairments and clear potential for bioaccumulative escalation underscore an urgent need for enhanced environmental monitoring, stringent regulatory enforcement, and targeted research, particularly in underrepresented regions, to safeguard public health and ecosystem integrity.

## 1. Introduction

Latin America and the Caribbean (LAC) is a region of immense ecological wealth, hosting some of the planet's most vital biodiversity hotspots and freshwater resources. However, this natural heritage is under escalating threat from anthropogenic pollution, which disrupts ecological balance and poses significant risks to human health [1–5]. Globally, pollution is a leading environmental cause of disease and mortality [6], with its burdens often falling disproportionately on marginalized and vulnerable communities [7–9]. The contamination of fresh water is a key part of this global concern, driven by the constant release of natural and anthropogenic substances, which demands a better knowledge of the chemical status of surface waters [10].

In LAC, soil pollution and water pollution present a paradoxical crisis. The region's fertile soils and pristine waterways are increasingly contaminated by activities such as intensive agriculture, mining, oil extraction, and rapid urbanization [11–15]. Water quality monitoring studies targeting different classes of substances are crucial to understand this impact, as performed in different regions worldwide [10]. Soil pollution, originating from industrial expansion and agrochemical overuse, degrades soil fertility, reduces crop yields, and introduces toxins into the food chain and adjacent water bodies [3, 16]. Concurrently, water pollution from urban sewage, industrial effluents, and agricultural runoff carries a complex mixture of pesticides, pharmaceuticals, and heavy metals into rivers, lakes, and coastal zones [17–21]. These contaminants not only are detrimental to aquatic life but also bioaccumulate in food webs, posing latent risks to human populations that rely on these ecosystems for food and water [22–24].

Despite the gravity of the situation, the evidence on the impacts of soil and water pollution across LAC remains fragmented. While numerous individual studies have documented contamination and its effects, a comprehensive synthesis is needed to identify consistent patterns, highlight regional research trends, and pinpoint critical knowledge gaps. Previous reviews have often focused on specific pollutants or subregions, but a broader perspective is required to inform regional policy and prioritize future research investments.

### 1.1. Objectives of This Review

To address this need, we conducted a systematic review to map and synthesize the available empirical evidence on the impacts of soil and water pollution on ecosystems and public health across LAC. The specific objectives were as follows.

1. To systematically identify and characterize peer-reviewed studies published between 2008 and 2023 that investigate soil and/or water pollution and its health or ecological effects in LAC countries.
2. To descriptively synthesize the findings regarding the types and concentrations of key contaminants (e.g., pesticides and heavy metals), their documented health impacts (e.g., subclinical effects and biomarker changes), and their ecological consequences.
3. To identify and analyze geographical and methodological trends in the existing research, highlighting well-studied areas and significant gaps, particularly in regions like Central America and the insular Caribbean.
4. To discuss the implications of the synthesized evidence for environmental monitoring, public health protection, and future research directions in the region.

By adopting this systematic, descriptive approach, this review aims to provide a clearer picture of the pollution landscape in LAC, offering a valuable evidence base for scientists, policymakers, and public health practitioners to address this pressing environmental challenge.

## 2. Methodology

To achieve the objectives of this systematic review, a rigorous and reproducible methodology was employed to identify, select, and synthesize the available evidence on soil and water pollution impacts in LAC. The approach was designed to minimize bias and provide a comprehensive qualitative synthesis of the literature.

### 2.1. Search Strategy and Information Sources

A systematic search was conducted across five major electronic databases: EBSCOhost, PubMed, SciELO, JSTOR, and HINARI. The search aimed to capture all relevant peer-reviewed literature published within a 15-year period from 2008 to 2023. The strategy was built upon a framework of key concepts related to the population, exposure, and outcomes of interest.

The search string combined keywords and Medical Subject Headings (MeSH) terms using Boolean operators. The core concepts included “water pollution” OR “soil pollution” OR “pesticides” OR “metals” OR “heavy metals” OR “ecosystem pollutants” OR “aquatic life pollutants.” This approach is aligned with global monitoring efforts that target specific classes of pollutants, including those identified as priority substances and contaminants of emerging concern [10]. These were combined with geographic terms: “Latin America” OR “Caribbean” OR “Central America” OR “South America.” To capture the context of impacts, terms such as “public health,” “health impacts,” “ecosystem health,” and “bioaccumulation” were also included. This strategy was designed to ensure broad coverage of studies linking contamination pathways, such as agricultural runoff and industrial discharge, to ecological and human health endpoints across the region's diverse landscapes, as highlighted in the existing literature [1, 3, 7].

### 2.2. Eligibility Criteria

Study eligibility was determined using predefined inclusion and exclusion criteria, detailed in Table 1. Included studies were required to be English-language, peer-reviewed articles presenting empirical research conducted within LAC countries, and published within the specified timeframe. The focus was on studies that provided quantitative data on soil or water pollution and its measurable impacts on human health biomarkers, ecological endpoints, or contaminant concentrations in environmental matrices. Exclusion criteria encompassed non-English publications, literature reviews, commentaries, studies conducted outside LAC, and purely qualitative or in vitro studies, as these do not reflect real-world exposure pathways.

### 2.3. Study Selection Process

**Figure 1:**
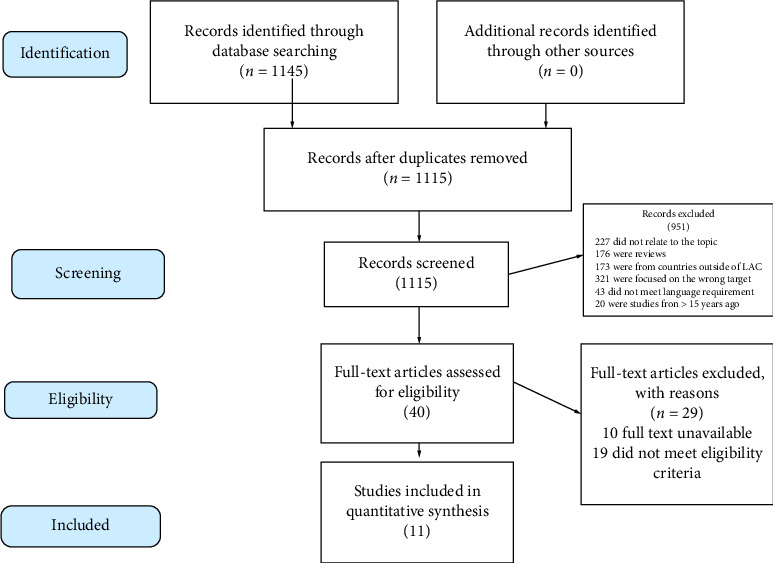
PRISMA flow diagram illustrating the systematic literature search, screening, and study selection process.

The study selection process followed a two-phase screening protocol to ensure consistency and minimize error. All records identified through the database searches (*n* = 1145) were imported into Rayyan, an AI-assisted systematic review software program, for management and blinded screening. The first phase involved a title and abstract screening against the eligibility criteria. In the second phase, the full texts of potentially relevant studies were retrieved and subjected to a rigorous evaluation for methodological quality, relevance, and sufficiency of data for extraction. The flow of studies through the identification, screening, eligibility, and inclusion stages is documented in a PRISMA-compliant flowchart (Figure 1).

### 2.4. Data Extraction and Management

Data from the final set of included studies were extracted into a standardized template. The extracted information encompassed several key domains: geographic context (specific country and region); study population or environmental setting; pollutant profiles (specific classes of pesticides, heavy metals, and their measured concentrations); exposure assessment methods (e.g., biomonitoring, environmental sampling of water, sediment, or biofilm); and reported health or ecological endpoints (e.g., biomarker levels, prevalence of symptoms, biodiversity metrics, and risk quotients). This structured extraction allowed for the systematic organization of evidence for subsequent synthesis.

### 2.5. Risk of Bias Assessment

The methodological robustness of individual studies was critically appraised through a risk-of-bias assessment conducted by an independent reviewer using Rayyan's blinded function. This assessment evaluated key elements such as the representativeness of sampling strategies, the analytical validity and detection limits of contaminant measurements, and the degree of control for potential confounding factors. This process informed the qualitative interpretation of the findings but was not used as a basis for exclusion, allowing for a comprehensive overview of the available evidence.

### 2.6. Data Synthesis

Given the heterogeneity in study designs, populations, exposures, and outcome measures identified—a finding consistent with the reviewer's feedback—a quantitative meta-analysis was deemed inappropriate. Instead, a descriptive synthesis approach was adopted to address the review's objectives. The synthesis involved a systematic narrative summary and thematic analysis of the extracted data. Studies were grouped and analyzed by geographic region, type of pollutant, and category of impact (human health vs. ecological). The findings are presented to illustrate trends, consistencies, and contradictions in the evidence and to clearly map the distribution of research activity across the LAC region. This method provides a transparent and critical overview of the current state of knowledge, effectively highlighting both the documented impacts of pollution and the significant gaps that remain in the scientific literature.

## 3. Results

### 3.1. Study Selection and Geographical Distribution

The systematic search across five electronic databases yielded 1145 records. Following a rigorous, two-phase screening process against predefined eligibility criteria, 11 studies were ultimately included for qualitative synthesis. The study selection process, detailed in the PRISMA flowchart (Figure 1), was characterized primarily by the exclusion of nonempirical studies, research conducted outside LAC, and investigations that lacked quantitative data on pollution impacts.

A critical finding of this review is the pronounced geographical skew in the distribution of the included studies. As detailed in Table 2, the evidence base is overwhelmingly dominated by research from Brazil, which contributed eight of the 11 studies. The remaining studies originated from Colombia (2) and Uruguay (1). This distribution reveals a substantial research gap, as our search identified no studies meeting the inclusion criteria from Central American nations or the insular Caribbean, despite the known pollution pressures from agriculture and urbanization in these regions [12, 13].

Thematically, the included studies fell into two broad categories. Three studies focused specifically on occupational pesticide exposure and its health effects in rural worker populations in Brazil [25–27]. The remaining eight studies quantified contaminant loads and assessed ecological risks within aquatic ecosystems, predominantly in agricultural zones of Brazil, with single studies from Colombia and Uruguay [19, 22–24, 28–31].

### 3.2. Synthesis of Health Impacts From Soil and Pesticide Exposure

The evidence from human studies consistently pointed to subclinical health impairments associated with pesticide exposure, rather than acute poisoning events. Among rural workers in Brazil, respiratory symptoms were a common finding. Buralli et al. [25] documented a prevalence of cough in 40% of participants during the crop season, persisting in 30.7% during the off-season, alongside notable rates of nasal allergies and chest tightness, linking these symptoms to proximity pesticide application.

Further evidence indicated disruptions to endocrine and neurological systems. Freire et al. [26] identified strong and statistically significant correlations between serum concentrations of organochlorine pesticides (OCPs) and altered levels of sex hormones in both men (*r* = 0.68, *p* < 0.001) and women (*r* = 0.72, *p* < 0.001) living in a contaminated rural area Similarly, Piccoli et al. [32] documented associations between pesticide exposure and altered thyroid function in an agricultural population in Brazil, further evidencing endocrine system disruption. Complementing this, Silvério et al. [27] reported evidence of cholinesterase inhibition, a marker of neurotoxicity, in 63.8% of workers exposed to organophosphates. The widespread exposure was further confirmed by the detection of dialkyl phosphate metabolites in the urine of 92.6% of the organophosphate-exposed group. It is important to note that none of the included studies reported acutely elevated serum pesticide concentrations at a population level, underscoring the chronic, low-dose nature of the documented exposures.

### 3.3. Synthesis of Aquatic Contamination and Ecological Risks

The studies monitoring aquatic environments revealed widespread contamination by pesticides and metals, with concentrations spanning several orders of magnitude across different environmental matrices. The data on measured concentrations, as extracted from the included studies, are summarized in Table 3.

Herbicides were frequently dominant in the contaminant profiles. Guarda et al. [19] detected the herbicide clomazone in 100% of surface water samples from the Formoso River, with concentrations reaching 0.538 μg/L. Similarly, studies in sugarcane cultivation areas, such as that by Acayaba et al. [22], identified a complex mixture of pesticides including imidacloprid, carbendazim, and tebuthiuron, the latter of which was also detected in groundwater at 107 ng/L. Kuhn et al. [30] reported the presence of imazethapyr and tebuconazole in tributaries of the Uruguay River.

Metal contamination was particularly documented in coastal systems. In the Gulf of Urabá, Colombia, Pemberthy et al. [24] measured chromium and lead in seawater, with lead concentrations ranging from 0.012 to 0.165 mg/L. More critically, this study and that of Gallego Rios et al. [31] demonstrated clear bioaccumulation, with lead and mercury being detected in the muscle tissue of commercially important fish species at levels posing potential human health risks.

Several studies conducted formal ecological risk assessments, with findings indicating latent but significant threats. Machado et al. [23] calculated that arsenic concentrations in the Pardo River presented a carcinogenic risk greater than 10^−6^, exceeding US EPA thresholds. Furthermore, Acayaba et al. [22] concluded that mixtures of herbicides, including ametryn, atrazine, and diuron, posed a definite risk to aquatic life in the studied regions.

### 3.4. Synthesis of Evidence and Identification of Gaps

The convergence of evidence from human and ecological studies paints a picture of pervasive, low-level contamination across the studied parts of LAC. The key findings signal a potential for bioaccumulative escalation, evidenced by the persistence of biomarkers of effect in human populations, the spatiotemporal patterns linking agricultural zones to aquatic pollutant loads, and the quantification of ecological risks from contaminant mixtures.

This synthesis, however, is constrained by significant gaps in the literature. The most evident gap is geographical, with a near-total absence of studies from Central America and the Caribbean. Furthermore, methodological heterogeneity in exposure assessment and outcome measurement limited the direct comparability of findings across studies. The evidence base is also characterized by a focus on a limited set of legacy pollutants and a scarcity of longitudinal data, which restricts the ability to assess trends in contaminant accumulation or the long-term progression of subclinical health effects.

## 4. Discussion

This systematic review synthesizes a body of evidence that reveals a consistent and concerning narrative of environmental contamination across LAC. The findings depict a scenario not of acute, catastrophic pollution events, but of a pervasive, insidious infiltration of agrochemicals and metals into ecosystems and human populations. The most immediate conclusion from our synthesis is the profound geographical bias in the research landscape; the overwhelming concentration of studies in Brazil, as detailed in Table 2, leaves the pollution status of vast and vulnerable regions like Central America and the insular Caribbean largely unknown and unmonitored. This evidence gap is particularly alarming given that these regions share similar pressures from agricultural intensification and urbanization, suggesting a potentially invisible public health and ecological crisis.

The human health evidence, drawn exclusively from Brazilian occupational cohorts, consistently demonstrates that the absence of acute poisoning does not equate to an absence of harm. The pervasive subclinical effects—ranging from respiratory morbidity [25] to significant endocrine disruption [26] and neurotoxic inhibition of cholinesterase [27]—collectively signal a state of chronic physiological stress in exposed populations. These findings align with a broader literature indicating that low-level, continuous exposure can disrupt homeostatic mechanisms long before clinical disease manifests. The strong correlations between organochlorine body burden and sex hormone alterations [26] are especially compelling, providing a plausible biological pathway for the reproductive health impacts observed in other studies, such as depressed hormone levels and compromised sperm quality in young rural men [33, 34]. This pattern of subclinical impairment underscores a latent risk, where the cumulative burden of exposure may precipitate more severe chronic diseases over time, as suggested by the association between pesticide usage and mortality from acute kidney failure [35].

Parallel to the human evidence, the environmental data paint a picture of widespread aquatic system compromise. The extreme contamination gradients summarized in Table 3, spanning from nanogram per liter concentrations in biofilms [28] to milligram per liter levels of metals in coastal waters [31], highlight the diverse and intense pollution pressures across LAC. The dominance of herbicides like clomazone, detected in 100% of river samples in one study [19], and the persistence of tebuthiuron in groundwater [22] point to a systemic issue rooted in modern agricultural practices. Furthermore, the detection of OCPs in estuary sediments at levels as high as 154.43 ng·g^−1^ [29] is a stark reminder of the enduring legacy of past pesticide use, creating persistent reservoirs of contamination that continue to leak into the environment.

The convergence of human and ecological evidence points squarely to the critical process of bioaccumulation. The detection of metals in fish muscle at concentrations higher than the surrounding water [24, 31] and the presence of OCPs and DDT metabolites in consumed fish species [36] complete a dangerous exposure pathway from contaminated environments to human food chains. This is the core of the latent risk identified in this review: the continuous input of pollutants, even at individually low concentrations, creates a scenario where bioaccumulation can elevate internal doses in biota to hazardous levels. The ecological risk assessments conducted within the included studies substantiate this threat, with herbicide mixtures identified as a danger to aquatic life [22] and arsenic levels in water exceeding carcinogenicity thresholds [23]. Therefore, the findings presented in Tables 2 and 3 should not be interpreted as a baseline of safety, but rather as a snapshot of a dynamic system with a clear potential for bioaccumulative escalation.

The pervasive nature of low-level contaminants, coupled with the evidence of their subclinical and ecological impacts, demands a paradigm shift in environmental monitoring and public health protection. The findings compel a move beyond a regulatory framework focused primarily on acute toxicity and individual compound limits, toward one that accounts for chronic, low-dose exposure to complex chemical mixtures. This need is recognized globally, as evidenced by regulatory efforts like the EU's Watch List, which aims to identify potential future priority substances based on monitoring data regarding their frequency and concentration [10]. The documented presence of these pollutants in breast milk [37, 38] further highlights the intergenerational implications of this continuous contamination, exposing the most vulnerable during critical developmental windows. In conclusion, the evidence synthesized in this review reveals that the threat of soil and water pollution in LAC is not diminishing but is instead evolving into a more subtle, chronic, and deeply embedded challenge that requires equally sophisticated and sustained countermeasures.

### 4.1. Recommendations

To mitigate the latent risks identified in this review and safeguard both ecosystem integrity and public health across LAC, a concerted, multipronged strategy is imperative. The following evidence-based recommendations are proposed.

First, the establishment of continuous, standardized environmental surveillance systems is paramount. These systems must be designed to quantify contaminant fluxes across the soil-water-biota continuum, utilizing harmonized EPA/WHO metrics to track spatial and temporal trends of priority pollutants. Such monitoring should be informed by global data on the most frequent and high-concentration substances, which can help identify regional priority contaminants and future candidates for regulation [10]. Monitoring must explicitly target known agricultural hotspots and industrial corridors but must also be strategically deployed to address the critical data gaps in underrepresented regions of Central America and the insular Caribbean. This will transform the current fragmented snapshot into a dynamic picture of regional contamination, enabling early detection of pollution incidents and informed resource allocation. Second, regulatory frameworks must be strengthened to enforce stricter controls at the source. This should include imposing more stringent restrictions on pesticide application practices and mandating advanced wastewater treatment technologies, such as closed-loop systems, for industrial point sources—particularly from mining and manufacturing sectors. Regulatory thresholds should be dynamically informed by real-time monitoring data and must increasingly account for the toxicological realities of chronic low-dose exposure and mixture toxicity, which current standards often overlook. The promotion of integrated pest management (IPM) strategies and agroecological transitions, incorporating buffer zones and precision agriculture, can substantially reduce the agrochemical load entering aquatic systems, thereby mitigating the risks documented in studies like those of Acayaba et al. [22] and Guarda et al. [19].

Third, research investments must be strategically directed. Longitudinal biomonitoring studies of vulnerable populations—including children, pregnant individuals, and subsistence fishing communities—are essential to quantify exposure pathways and elucidate the long-term consequences of the subclinical health impacts, such as endocrine disruption and cholinesterase inhibition, identified in this review. Concurrently, ecotoxicological research must prioritize characterizing the bioaccumulation kinetics of contaminant mixtures across trophic levels, using approaches like species sensitivity distribution (SSD) to inform evidence-based revisions of ecological risk thresholds. Finally, regional policy coordination through transboundary initiatives is crucial to standardize regulatory enforcement, facilitate knowledge transfer, and transform scientific evidence into actionable, region-wide governance.

### 4.2. Limitations

This systematic review acknowledges several methodological constraints that necessarily impact the interpretative scope and generalizability of its findings. The most significant limitation is the pronounced geographical imbalance within the evidence base. With 8 of the 11 included studies originating from Brazil, the synthesis is inherently skewed and cannot fully represent the diverse agroecological, industrial, and socioeconomic contexts found across the entire LAC region. This regional bias may obscure unique pollution dynamics and exposure pathways in underrepresented areas, such as Central America and the Caribbean, which may be experiencing rapid and unmonitored environmental degradation.

Further constraints arise from methodological heterogeneity across the included studies. Variability in exposure assessment protocols—including differing biomonitoring matrices (serum and urine), environmental sampling methodologies (water column vs. biofilm), analytical techniques, and detection limits—impeded direct comparability and contributed to the high clinical diversity that precluded meta-analysis. The predominance of cross-sectional study designs provides only a snapshot in time, restricting our ability to assess longitudinal trends in contaminant accumulation or the progression of subclinical health effects to manifest chronic disease.

Furthermore, the focus of the included studies on a limited set of pollutant classes, notably organochlorines, organophosphates, and priority metals, may overlook the risks posed by emerging contaminants and the complex synergistic interactions of chemical mixtures in real-world environments [39]. The heavy reliance on occupational cohorts for human health endpoints, while valuable, limits the generalizability of findings to the general population, particularly more vulnerable subgroups such as children or communities reliant on subsistence fishing. These constraints collectively underscore the urgent need for more standardized, regionally harmonized, and longitudinal research frameworks to strengthen future evidence synthesis on pollution impacts across LAC.

### 4.3. Conclusions

This systematic review synthesizes empirical evidence demonstrating that the impact of soil and water pollution across LAC manifests primarily through subclinical and chronic pathways, creating a state of latent ecological and public health risk. The evidence reveals a consistent pattern of widespread low-level contamination, characterized by pervasive biomarker evidence of subclinical health impairment in human populations and significant contaminant loads across diverse aquatic ecosystems. The ubiquity of herbicides and the clear bioaccumulation potential of heavy metals underscore a progressive environmental infiltration that, while not yet statistically significant in acute terms, establishes a dangerous foundation for future escalations.

The convergence of findings—encompassing human biomarker persistence, spatiotemporal contamination patterns, and ecological risk quotients exceeding safety thresholds—signals a clear and present danger. The absence of acute statistical significance in pooled estimates is more indicative of regional data disparities and methodological heterogeneity than an absence of risk. The identified potential for bioaccumulative escalation demands a proactive and vigilant response. Consequently, the implementation of continuous environmental monitoring, the enforcement of stringent, forward-looking regulations, and the strategic pursuit of research aimed at filling critical knowledge gaps are not merely recommendations but imperative actions. Therefore, disrupting the cycle of bioaccumulation through the recommended measures is not merely an environmental management goal but an urgent public health imperative. Proactive, science-driven stewardship is essential to safeguard ecosystem integrity and protect vulnerable populations across the LAC region, preventing the current state of latent risk from escalating into a full-blown crisis.

## Figures and Tables

**Table 1:** Eligibility criteria for the systematic review on soil and water pollution impacts in Latin America and the Caribbean.

Inclusion criteria	Exclusion criteria
English-language peer-reviewed articles	Non-English publications
Empirical research designs	Literature reviews, commentaries
Published between 2008 and 2023	Studies published > 15 years ago
Conducted in LAC countries	Studies outside LAC
Quantitative exposure/outcome data	Qualitative or non-data-driven studies
Focus on soil/water pollution impacts	Laboratory-only (*in vitro*) research

**Table 2:** Characteristics and geographical distribution of the 11 studies included in the systematic review.

Reference	Country	Country income	Research aim	Exposure assessment	Outcome
Buralli et al. [25]	Brazil	Upper middle income	The objective was to evaluate whether pesticide exposure is associated with respiratory outcomes among rural workers and relatives in Brazil during the crop and off-seasons.	Spirometry	During the crop and off-season, participants presented a prevalence of 40% and 30.7% for cough, 30.7% and 24% for nasal allergies, and 24% and 17.3% for chest tightness.
Freire et al. [26]	Brazil	Upper middle income	To examine the association between serum concentrations of OC pesticides and levels of sex hormones in the adult population in a rural area in Brazil heavily contaminated with pesticides	A cross-sectional study with 304 men and 300 women was undertaken. Wet-weight serum concentrations of 19 OC pesticides were determined in all participants.	The compounds showing the highest prevalence and serum levels were *r* = 0.68 in males (*p* < 0.001) and *r* = 0.72 in females (*p* < 0.001).
Silvério et al. [27]	Brazil	Upper middle income	The study aimed to assess occupational exposure to pesticides in rural workers using genotoxicity tests, bioindicators, and clinical evaluation.	Samples (buccal, urine, and blood) from rural workers exposed to pesticides (with organophosphates and without organophosphates) were compared with the activities of urinary dialkyl phosphates, cholinesterases, and genotoxicity data from a cytometry assay.	The group exposed to organophosphates had levels of acetylcholinesterase (63.8%), butyrylcholinesterase (12.8%), and total cholinesterase (14.8%), with 92.6% having dialkyl phosphates present in their urine.
Acayaba et al. [22]	Brazil	Upper middle income	This study aimed to analyze 13 pesticides and one degradation product in surface and groundwater in the region with the most significant sugarcane production in the world.	Multiresidue method	For the surface water, 2-hydroxyatrazine, diuron, carbendazim, tebuthiuron, and hexazinone were the most frequently detected (100%, 94%, 93%, 92%, and 91%, respectively). Imidacloprid (2579 ng·L^−1^), carbendazim (1114 ng·L^−1^), ametryn (1101 ng·L^−1^), and tebuthiuron (1080 ng·L^−1^) were found at the highest concentrations. For groundwater, tebuthiuron was the only quantified pesticide (107 ng·L^−1^). Ametryn, atrazine, diuron, hexazinone, carbofuran, imidacloprid, malathion, carbendazim, and their mixtures presented a risk for aquatic life.
Rheinheimer et al. [28]	Brazil	Upper middle income	This study identified and quantified pesticides in epilithic biofilms to evaluate this matrix's effectiveness.	Bioindicators and biofilms were utilized.	The maximum concentrations of herbicides in biofilms were 17.6, 445.9, 22.3, and 59.6. The highest concentrations of insecticides in biofilms were 20.3 and 14.3. Finally, the highest concentrations of fungicides in biofilms were 73.0, 44.6, and 35.9.
Oliveira et al. [29]	Brazil	Upper middle income	This study evaluated the legacy of banned OCP usage, considering the levels, ecological risk, and dependence on sediment physicochemical properties that affect the fate and distribution of the Jaguaribe River.	The sum concentration of OCPs (ΣOCPs) ranged from 5.09 to 154.43 ng·g^−1^	The sum concentration of OCP ranged from 5.09 to 27.38 ng·g^−1^ (fluvial zone) and from 79.45 to 154.43 ng·g^−1^ (estuary zone). S7 station (estuary) and S3 station (river) had the highest OCPs, 154.43 ng·g^−1^ and 27.38 ng·g^−1^
Machado et al. [23]	Brazil	Upper middle income	This study aimed to assess the human health risks of environmental exposure to herbicides and metals through water and fish intake in the Pardo River.	Metals were analyzed in river water and edible fish. Herbicides were analyzed in river water.	Al, Cd, Cu, Mn, Pb, and Zn levels in river water exceeded US EPA benchmarks. Noncarcinogenic risks due to pollutant mixture exposure were above the limit, and carcinogenic risks of As were as follows: Exposure was > 10^−6^ in the sampling points during the rainy season. Herbicides were detected in four sampling points, with atrazine concentrations (range 0.16–0.32 μg/L) below the Brazilian standard (2.0 μg/L) but above the European Union standard (0.1 μg/L).
Guarda et al. [19]	Brazil	Upper middle income	Thirty-one active ingredients of pesticides of different classes were analyzed.	UHPLC-MS/MS quarterly collections in the region's dry and rainy seasons helped evaluate the impact of pesticides on the study site's biodiversity.	The active ingredient clomazone was present in all trial points, with concentrations reaching up to 0.538 μg·L^−1.^
Kuhn et al. [30]	Uruguay	Upper middle income	This study analyzed the physicochemical characteristics, metals, and pesticide levels in water samples obtained before and after the planting and pesticide application season from three sites: Uruguay River and two minor tributaries, Mezzomo Dam and Salso Stream.	They analyzed the physicochemical characteristics, metals, and pesticide levels in water samples obtained.	The highest clomazone concentrations found in the tested water samples were 0.00176 mg/L, 0.0248 mg/L for imazethapyr, and 0.00366 mg/L for tebuconazole.
Pemberthy et al. [24]	Gulf of Urabá, Colombian Caribbean	Upper middle income	The study evaluated the contents of chromium (Cr), lead (Pb), and mercury (Hg) in seawater and fish muscle in three fish species from the Gulf of Urabá that are commercialized and consumed by the population of the municipality of Turbo, using microwave-induced plasma optical emission spectrometry (MIP OES).	Microwave-induced plasma optical emission spectrometry (MIP OES).	Cr and Pb concentrations in seawater from several sampling points ranged from 0.025 to 0.369 mg/L and 0.012–0.165 mg/L, respectively, while Hg levels were below the detection limit. Regarding fish samples, Pb and Hg levels range from 0.64–1.91 mg/kg and 0.11–1.09 mg/kg, respectively. Sea catfish species exhibited the highest content of metals, followed by stone head catfish and anchovy.
Gallego Rios et al. [31]	Gulf of Urabá, Colombian Caribbean	Upper middle income	concentrations of Hg, Pb, and Cd in the muscle and waste material (head-gills, viscera, and fin-tail) of one of the most commercially important species, the crevalle jack (*Caranx hippos*), to determine their presence in body parts used in the production of subproducts	Hg, Pb, and Cd levels were compared between the waste material and muscle of the crevalle jackfish caught from the following sites the community usually uses to catch them.	

**Table 3:** Measured concentrations of pesticides and metals in aquatic systems of Latin America and the Caribbean, illustrating a gradient spanning several orders of magnitude.

Study	Experimental mean (μg/L)	Experimental SD	Experimental total
Acayaba et al. [22]	0.0054	0.001465	1
Rheinheimer et al. [28]	0.0047	0.0111	2
Oliveira et al. [29]	0.01367	0.0298	2
Machado et al. [23]	0.065	0.0672	3
Guarda et al. [19]	0.3024	0.1004	4
Kuhn et al. [30]	6.5079	11.2304	1
Pemberthy et al. [24]	1600.71	1865.0071	2
Gallego Rios et al. [31]	2110	2529.12	1

*Note:* Experimental total: number of measurement points/samples per study.

## Data Availability

All the data generated and associated with this research have been provided in this article.
